# A large outbreak of giardiasis in a municipality of the Bologna province, north-eastern Italy, November 2018 to April 2019

**DOI:** 10.2807/1560-7917.ES.2021.26.35.2001331

**Published:** 2021-09-02

**Authors:** Davide Resi, Stefania Varani, Anna Rosa Sannella, Alessandra M De Pascali, Margherita Ortalli, Giovanna Liguori, Marco Benvenuti, Maria C Re, Roberta Pirani, Luciana Prete, Claudia Mazzetti, Muriel Musti, Lorenzo Pizzi, Tiziana Sanna, Simone M Cacciò

**Affiliations:** 1 Unit of Hygiene and Public Health, Department of Public Health, Bologna, Italy; 2 Department of Experimental, Diagnostic and Specialty Medicine, University of Bologna, Italy; 3 Unit of Microbiology, IRCCS Azienda Ospedaliero-Universitaria di Bologna, Bologna, Italy; 4 Unit of Foodborne and Neglected Parasites, Department of Infectious Diseases, Istituto Superiore di Sanità, Rome, Italy; 5 Unit of Nutrition, Diet and Lifestyle, Department of Public Health, Bologna, Italy; 6 Unit of Epidemiology, Health Promotion and Risk Communication, Department of Public Health, Bologna, Italy

**Keywords:** outbreak, water, Giardia, Italy

## Abstract

Giardiasis, the disease caused by the flagellate *Giardia duodenalis* (syn. *G.lamblia, G. intestinalis*), is the most commonly reported among the five food- and waterborne parasitic diseases under mandatory surveillance in 24 EU countries. From November 2018 to April 2019, an outbreak of giardiasis occurred in a municipality of the Bologna province, in north-eastern Italy. Microscopy and immunochromatography identified cysts and antigens, respectively, of the parasite in stool samples of 228 individuals. Molecular typing of 136 stool samples revealed a vast predominance (95%) of *G. duodenalis* assemblage B. Investigations into potential sources indicated tap water as the most likely vehicle of infection, although cysts were not detected in water samples. Control measures mostly aimed at preventing secondary transmission by informing citizens about the outbreak, and by treatment of patients with anti-parasitic drugs. This is the first documented human outbreak of giardiasis in Italy; its investigation has highlighted the difficulties in the timely detection and management of this parasite, which is often overlooked as a cause of human gastroenteritis. The long and variable incubation time, absence of specific symptoms and a general lack of awareness about this pathogen contributed to delay in diagnosis.

## Background

The unicellular flagellated parasite *Giardia duodenalis* (synonyms, *Giardia lamblia, Giardia intestinalis)* infects the gastrointestinal tract of a wide range of mammals [1]. In humans, giardiasis is caused by assemblages A and B, two genetically different groups that have zoonotic potential [2]. The simple life cycle comprises the trophozoite, which causes the symptoms, and the cyst, the infective stage, which is shed with the host’s stools.

Transmission occurs by the faecal-oral route or by ingestion of cysts in contaminated water or food [1]. Water plays an important role in the transmission of *Giardia* infection; the cysts survive best in cool, damp environments and can withstand chlorination, maintaining their infectivity for weeks. Waterborne outbreaks of giardiasis, indeed, have occurred worldwide [3]. In Europe, these have been mainly reported by Nordic countries, including a large outbreak in Bergen, Norway, which occurred in 2004 and involved around 6,000 individuals [4].

Giardiasis is the most reported infection among the five food- and waterborne parasitic diseases under mandatory surveillance in 24 European Union (EU) countries. However, Italy, Austria, Denmark, France and the Netherlands do not currently have a compulsory reporting system [5]. In the 2017 annual epidemiological report from the European Centre for Disease Prevention and Control (ECDC) [5], 19,437 confirmed giardiasis cases were reported by 24 countries in the EU/European Economic Area (EEA), with an overall rate of 5.5 cases per 100,000 inhabitants. The highest notification rate was in the age group 0–4 years, namely 17.6 cases per 100,000 inhabitants for males and 14.9 for females.

### Outbreak detection

On 8 January 2019, the Parasitology section of the Microbiology Unit, University Hospital of Bologna, Bologna, Italy (ParaLab-Bo) identified 10 cases of *Giardia* infection. Considering the extraordinary number of cases diagnosed within one day, the medical parasitologist decided to inform the local public health office (LPHO). The ParaLab-Bo serves as the public laboratory for the identification of intestinal parasites in the Bologna province. All samples were from the ‘Pianura Ovest’ health district and all were from residents of a municipality of 7,387 inhabitants (Municipality A) within the metropolitan city of Bologna, north-eastern Italy. General practitioners (GPs) reported three additional cases from the same municipality between 27 December 2018 and 2 January 2019. In comparison, only one case was reported in the entire ‘Pianura Ovest’ health district from January to October 2018.

The first case interviews indicated consumption of tap water as the most likely source of infection, while involvement of a common food item was excluded. No history of recent travel abroad was reported by any cases. Therefore, in agreement with the definition by the European Food Safety Authority [6], an outbreak of giardiasis was declared in Municipality A on 8 January 2019. A task force, including representatives from the authorities of Municipality A, the Public Health Department of Bologna (PHD-Bo), ParaLab-Bo, and the Unit of Food-borne and Neglected Parasites at the Istituto Superiore di Sanità of Rome (UFNP-Rome), was established to investigate the source of the outbreak and to apply control and preventive measures. Here, we report on the investigation of this outbreak.

## Methods

### Epidemiological investigation

An outbreak-associated confirmed case of giardiasis was defined as a person who (i) met clinical and laboratory criteria for giardiasis [7], (ii) had onset of symptoms between 20 November 2018 to 20 April 2019, (iii) visited Municipality A at least two times within 1 week during the incubation period (estimated 1 to 4 weeks before the onset of symptoms [8]) and (iv) had not travelled abroad during the incubation period. An active surveillance was implemented each day from 9 January to 20 May 2019, and ParaLab-Bo reported all newly confirmed cases in residents from the health district ‘Pianura Ovest’ to the LPHO. GPs and paediatricians were informed about the outbreak and encouraged to submit samples from patients presenting with intestinal symptoms.

All but one of the confirmed cases (198/199) were invited for a telephone interview using a semi-structured questionnaire; the response rate was 99.8% (196/198). Data from infants and children (up to 14 years of age) were collected through their parents/custodians. In addition to demographic data and clinical information, the questionnaire explored the following domains: (i) food exposure (consumption of vegetables and fruits, stores where vegetables and fruits were purchased), (ii) water exposure (type of drinking water, accidental ingestion of untreated water, participation in water sports and other activities), (iii) animal exposure, (iv) travel history, (v) contact with other potential cases, (vi) gardening and (vii) attendance at day care, schools, public or private catering events. In addition, data on cases being treated were extracted from the local prescription database. The places where cases of *Giardia* infection resided were mapped and attack rates were calculated by street.

### Case–control study

An unmatched case–control study was performed to investigate potential source(s) of exposure. The sample size was calculated based on the primary hypothesis that there were no differences in tap water consumption between cases and controls. In the initial epidemiological investigation, we found that 92% of the individuals infected by *Giardia* drank tap water, while an estimated tap water consumption of 72%, taken from published studies of the Italian National Institute of Statistics, was used for the general population [9]. Based on these data, the sample size was calculated for a two-sided confidence level (1-alpha) of 95, a power (% chance of detecting) of 80, a hypothetical proportion of controls with exposure of 72%, and a hypothetical proportion of cases with exposure of 92%. The calculation of the sample size was obtained using OpenEpi, version 3, open source calculator (https://www.openepi.com/SampleSize/SSCC.htm) and the results were rounded up to the nearest integer. Accordingly, 60 cases were extracted randomly from the pool of cases, while 60 controls were randomly selected from individuals who fulfilled the following criteria: (i) had a stool sample negative for *G. duodenalis* that was provided for analysis between 20 November 2018 and 28 February 2019, (ii) resided in Municipality A or visited this municipality at least two times within 1 week during the estimated exposure period and (iii) had no history of travel abroad during the estimated exposure period. The exposure period considered for both cases and controls was between 12 November and 2 December 2018. Individuals who had gastrointestinal symptoms or who had been in contact with people with enteric symptoms or diagnosed with giardiasis during the outbreak were excluded from the control group. Among cases, some were clustered within the same household, namely four individuals in one cluster, and two individuals in each of six additional clusters.

Cases and controls were asked to report on exposures and risk factors during the estimated exposure period using a structured questionnaire that was administered by telephone and focused on the following risk factors: (i) consumption of tap water (number of glasses per day), (ii) consumption of water from drinking fountain, (iii) consumption of bottled water, (iv) use of domestic water filters, (v) contact with animals, (vi) gardening activities or cultivating a vegetable garden at home, (vii) swimming in pools, (viii) consumption of raw vegetables and fruits (number of servings per week) and (ix) residing on a street with high attack rate for *Giardia* infection, defined as a street with an attack rate higher than the 75th percentile (> 4.35 cases per 100 residents).

### Parasitological investigation

Infection was diagnosed by microscopy on stool samples stained with Lugol’s iodine, and by the ImmunoCard STAT! *Cryptosporidium*⁄*Giardia* immunochromatographic rapid assay (Meridian Bioscience, Inc., Cincinnati, Ohio, United States (US)) [10].

### Molecular characterisation

A total of 233 stool samples from 225 individuals were sent to the UFNP-Rome; of these, 13 were discarded due to an insufficient amount of material. To characterise the parasite, DNA was extracted from 220 stool samples using the FastPrep-24 instrument and the FastDNA SPIN Kit for Soil (MP Biomedicals, Solon, Ohio, US), and submitted to a PCR for amplification of the *beta-giardin* gene [11]. Positive PCR products were sequenced on both strands and *Giardia* assemblages and genotypes identified by Basic Local Alignment Search Tool (BLAST) searches (http://blast.ncbi.nlm.nih.gov/Blast.cgi). In a limited number of samples (n = 12), *G. duodenalis* assemblages were identified by restriction fragment length polymorphism (RFLP) analysis [11].

### Environmental investigations

**Figure 1:**
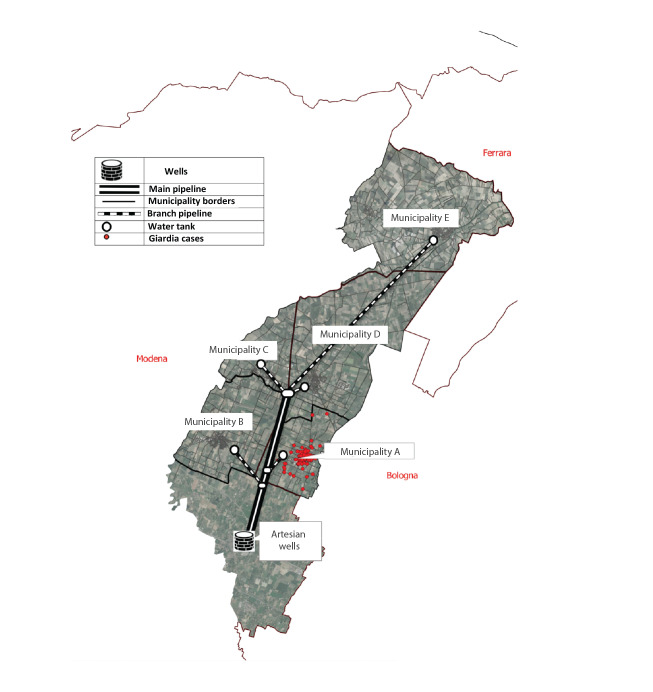
Map of the water supply system that serves Municipality A, Bologna province, north-eastern Italy, November 2018–April 2019

The scheme of the water supply system that serves Municipality A is depicted in Figure 1.

The geographical localisation of cases of giardiasis (red dots) is shown.

The municipal aqueduct of Municipality A is fed by the waterworks (Artesian wells, Figure 1). The collected water receives a light disinfection treatment with chlorine dioxide. A battery of pumps draws water from the collection tank and feeds it into the adduction duct (main pipeline, Figure 1) that serves five distinct municipalities, including Municipality A, by independent and terminal pipelines (branch pipeline, Figure 1).

On 9 January 2019, following the first reports of *Giardia* infection, samples were collected in Municipality A from the water tank (one sample), from a water house (two samples) and from the distribution pipeline (one sample in the central area of the municipality). Water samples (1 L each) were sent to the Istituto Zooprofilattico Sperimentale della Lombardia e Emilia Romagna (IZS) in Brescia, Italy and investigated for the presence of *G. duodenalis* by detection of DNA in concentrated water samples, according to a published method [12].

On 11 January 2019, additional samples were collected from the water tank in Municipality A from the distribution pipeline and from the plant of the artesian wells. Three water samples (10 L each) were collected and sent to the UFNP-Rome, where the presence of *Giardia* cysts was assessed by the standard method (International Organization for Standardization (ISO) 15553:2006 [13]).

After recognition of the outbreak, the managing body and the local authorities examined the data from the routine water analysis of the previous year. In accordance with the European Drinking Water Directive, these data included chemical-physical parameters, microbiological parameters, and phytosanitary residues measurement values [14]. As the Italian drinking water regulations do not include mandatory monitoring for *G. duodenalis*, the managing body of the water service implemented an additional monitoring plan of the Municipality A water network over a period of 150 days, starting from January 2019. Samples (1 L, n = 20) were investigated for the presence of *G. duodenalis* DNA [12] by IZS. Finally, plumbing works on the water distribution system that occurred during the suspected exposure period were mapped on the water network map and compared with the map of giardiasis cases by place of residence of confirmed cases. Data on local weather conditions during the probable exposure period were obtained from the regional agency for environmental and energy protection (Agenzia Prevenzione Ambiente Energia Emilia Romagna; Arpae-Simc., https://simc.arpae.it/dext3r).

### Data analysis

Statistical analyses were performed using the statistical package STATA Intercooled for Windows (version 12, STATACorp, College Station, Texas, US). Descriptive statistics (means, standard deviation (SD), percentage absolute frequencies, percentage relative frequencies) were calculated to describe the socio-demographic characteristics and risk factors. For the case–control study, risk factors, selected based on the epidemiological survey, were compared between cases and controls using univariate and multivariate logistic regression analyses to determine variables independently associated with giardiasis. The results were reported as odds ratios (OR) with 95% confidence intervals (CI) and two-tailed p values. The p values below 0.05 were considered significant. In order to ensure anonymity, each participant in the study was identified by a progressive alphanumeric identification code that could not be traced back to the identity of the person. Data were stored on a password-protected Excel-based database accessible only by the dedicated LPHO staff.

### Ethical statement

The study protocol was approved by the Ethics Committee of the Regional Health Authorities of Emilia-Romagna (Area Vasta Emilia-Centro, prot.nr. 71C; 28/04/20).

## Results

### Epidemiological, demographic and clinical presentation

**Figure 2:**
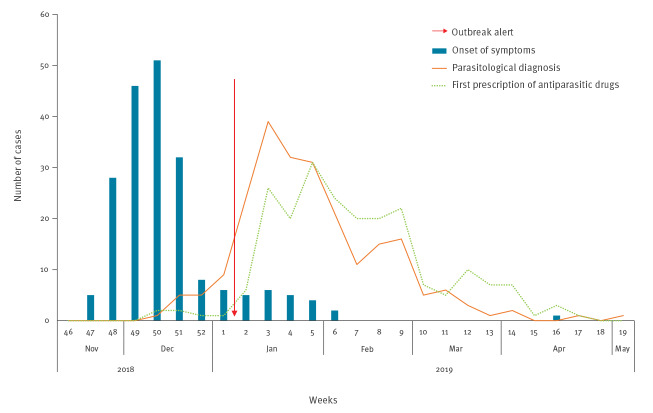
Epidemic curve of giardiasis cases in Municipality A, Bologna province, north-eastern Italy, November 2018–May 2019 (n = 228)

The first case of giardiasis was notified on 27 December 2018 (week 52); symptom onset was estimated to be around 11 December (week 50). Two additional cases were reported by GPs within week 1 in 2019 in individuals living in the same municipality. Between week 51 in 2018 and week 2 in 2019, 10 additional cases were diagnosed by ParaLab-Bo, but were either notified to the LPHO only after the identification of the outbreak (seven cases) or were not reported to the LPHO (three cases). The first 4 cases diagnosed by ParaLab-Bo had a clinical onset between 20–23 November 2018 (week 47), with a rapid increase in the following weeks; up to 80% of cases fell ill between 28 November (week 48) and 25 December (week 52). The outbreak reached its peak in week 50, then gradually declined. The last case, likely due to secondary transmission, was reported on 8 May 2019 (week 19). Based on the epidemic curve (Figure 2), the likely period of exposure for most cases spanned weeks 46 to 48 in 2018.

Blue histogram bars: number of cases by date of clinical presentation. Orange line: number of cases by time of parasitological diagnosis. Green dotted line: day when anti-parasitic drugs were first prescribed.

The average interval of time between onset of illness and diagnosis was 42 days (range: 2–125) and the outbreak onset was estimated 49 days before its identification. During the active surveillance period, 228 individuals tested positive for *G. duodenalis* cysts or antigens in the stool. Of these, 199 patients (87%) met the clinical and microbiological criteria for classification as confirmed cases of giardiasis, while 29 individuals (13%) were defined as asymptomatic carriers. Out of the 199 confirmed cases, 8 (4%) were not residents of Municipality A, but visited the municipality frequently for family, work or recreational reasons. Table 1 shows the sex and age distribution of cases and the specific attack rates.

**Table 1:** Sex and age distribution of cases and specific attack rates of giardiasis in Municipality A, Bologna province, north-eastern Italy, November 2018–May 2019 (n = 228)

Age group (years)	Sex distribution of cases						Specific attack rates (cases/100 inhabitants)		
	Female		Male		Total		Female	Male	Total
	n	%	n	%	n	%			
0–4	1	0.9	3	3.4	4	2.0	0.9	2.4	1.7
5–9	2	1.8	5	5.7	7	3.5	1.0	2.4	1.7
10–14	4	3.6	8	9.2	12	6.0	1.9	3.3	2.6
15–19	9	8.0	12	13.8	21	10.6	4.3	5.9	4.8
20–44	34	30.4	28	32.2	62	31.2	2.8	2.4	2.7
45–64	43	38.4	22	25.3	65	32.7	3.9	1.9	2.8
≥ 65	19	17.0	9	10.3	28	14.1	2.2	1.3	1.8
Total	112	56.3	87	43.7	199	100	2.9	2.3	2.6

The mean age of the confirmed cases was 40.6 ± 20.5 years. Overall, the attack rate was 2.6 per 100 inhabitants. The male-to-female case attack rate ratio was 0.7:1. Individuals aged 15–19 years had the highest age-specific incidence rate (4.8 per 100 inhabitants) compared with other age groups. 

The most frequently reported symptom was diarrhoea (87.4%; n = 174), followed by abdominal cramps (56.3%; n = 112), nausea or vomiting (55.3%; n = 110), signs of malabsorption (54.8%; n = 109) and abdominal bloating (39.7%; n = 79). Of the total, 116 cases (59%) had more than two symptoms and two patients required hospitalisation. Nearly all cases (96.5%; 193/199) were treated with anti-parasitic drugs. The two most common first-line treatments were metronidazole (50.3%; n = 100) and tinidazole (46.2%; n = 92). 

Among the potential risk factors investigated, drinking tap water from the local municipal supply (93.5%; 186/199) and having a close contact with individuals diagnosed with giardiasis (55.3%; 110/199) showed the most frequent association with the infection.

### Results from the case–control study

The response rate to the structured questionnaire was 98.3% for cases (59/60) and 100% for controls (60/60), therefore the overall response rate was 99.2%. A multivariate analysis indicated that drinking tap water was independently associated with *Giardia* infection, and that infection was strongly correlated with the number of glasses consumed daily (Table 2).

**Table 2:** Multivariate analysis of risk factors potentially associated with the *Giardia* outbreak from a case–control study, Municipality A, Bologna province, north-eastern Italy, November 2018−May 2019 (n = 59 cases, n = 60 controls)

Risk factor	OR	CI 95%		p value
Sex, female	1.78	0.56	5.67	0.33
Age (years)	0.99	0.95	1.02	0.39
Comorbidities	1.00	0.21	4.70	0.99
Consumption of tap water (n of glasses/day)^a^	1.98	1.40	2.80	< 0.01
Consumption of water from drinking fountain	4.03	0.60	27.29	0.15
Consumption of bottled water	1.91	0.43	8.40	0.39
Use of domestic water filtration systems	0.11	0.02	0.51	0.01
Contact with animals/owning pets	2.12	0.66	6.78	0.21
Gardening activities/cultivating a vegetable garden at home	4.44	1.14	17.29	0.03
Attending swimming pool	1.24	0.28	5.58	0.78
Weekly consumption of raw vegetables and fruits (n of servings/week)^b^	0.25	0.13	0.47	< 0.01
Residing in a high attack rate street	4.07	1.01	16.38	0.05

CI: confidence interval; OR: odds ratio.

^a^ OR was calculated for each additional glass of tap water per day.

^b^ OR was calculated for each additional serving of raw vegetables and fruits per week.

In fact, cases consumed an average of five glasses of tap water per day, whereas controls consumed an average of 2.70 glasses per day; for each additional glass of tap water per day, the probability of infection doubled (OR 2.0; 95% CI: 1.4–2.8). Moreover, gardening activities, cultivating a vegetable garden at home, and residing in a high attack rate street were associated with higher odds of giardiasis (OR: 4.4; 95% CI: 1.1–17.3 and OR: 4.1; 95% CI: 1.0–16.4, respectively). Conversely, those who used domestic water filters had reduced odds of giardiasis (OR: 0.1; 95% CI: 0.0–0.5). Consumption of raw vegetables and fruits was also associated with lower odds of acquiring giardiasis, with the probability of infection decreasing by 70% for each additional serving per week (OR: 0.3; 95% CI: 0.1–0.5). Other variables included in the multivariate analysis did not differ significantly between cases and controls (Table 2).

### Diagnostics and molecular characterisation of the parasite

During the outbreak, 1,215 faecal samples from 854 individuals residing in Municipality A or its surroundings were examined. Cases of *Giardia* infection were initially identified by microscopy (23 patients), then by a combination of microscopy and immunochromatography (35 patients), and finally by immunochromatography only (170 patients).

Of the 220 samples that underwent molecular investigation, 155 (70%) were amplified by the *beta-giardin* PCR assay. Of these, 19 (12%) were weakly amplified and could not be further analysed, while 12 (7.8%) were analysed by PCR-RFLP and yielded the pattern corresponding to assemblage B. Sequencing of the remaining 124 (80%) samples revealed assemblage B in 117 (94%) samples and assemblage A in seven samples (6%). Three distinct assemblage B genotypes were found in 76 (65%), 27 (23%) and 14 (12%) samples, respectively. For assemblage A, genotypes A1 (one sample), A2 (four samples) and A3 (two samples) were identified. Therefore, the vast majority (95%; 129/136) of the cases were infected by assemblage B. No mixed infections were identified.

### Results of environmental investigations

*Giardia* cysts and faecal coliform bacteria were not detected in any of the water samples collected from the distribution network on 9 and 11 January 2019. *Giardia* contamination was also not detected in any of the 20 samples obtained during the additional monitoring plan of the water supply network.

A review of the data collected during the year preceding the outbreak showed no contamination of the water network by sewage from residential areas, industrial activities or farms. During the estimated period of exposure, several instances of plumbing maintenance of the water supply network were documented. The spatial analysis did not reveal any association between the location of these works in Municipality A during the exposure period, and the streets where giardiasis cases resided. A temporal analysis could not be performed, as the exact timing of the operations was not available. No heavy rainfall occurred during the estimated exposure period, nor in the period immediately before this.

### Outbreak control measures

After recognition of the outbreak, a rapid response was achieved by the coordinated action of the ParaLab-Bo, PHD-Bo and UFNP-Rome. As *Giardia* contamination was not detected in the water samples analysed and the chemical-physical and microbiological standards were compliant with current legislation, a water avoidance notice was considered unnecessary by the authorities. Therefore, control measures aimed mostly at reducing secondary transmission. The municipality published two documents (on 12 and 26 January 2019) to inform the population about the outbreak and to disseminate the recommendations issued by the PHD-Bo. A dedicated webpage was created with informative material and a Frequently Asked Questions (FAQ) section, and an email address allowed submission of questions to experts.

An informative letter was sent to primary and secondary schools, kindergartens and nursing homes. Information about the transmission of *G. duodenalis* was provided to infected patients to minimise secondary transmission to close contacts, and to employees in food industries and healthcare who, in case of a positive diagnostic test, were instructed not to return to work for 48 hours following completion of treatment and resolution of symptoms. Inspections were carried out in restaurants, beauty salons, and schools to verify the management of hygienic conditions and provide information to protect customers and staff. PHD-Bo held a meeting with local GPs and paediatricians to share information on the diagnostic workflow and the specific treatments for giardiasis. The outbreak was declared resolved on 17 May 2019.

## Discussion

Waterborne outbreaks, which are caused by different bacterial, viral, and parasitic pathogens, are still a global public health concern [15]. Despite important advances in water management and sanitation, inactivation and/or removal of pathogens with resistant transmission stages, including parasites, is still challenging. This is the case for *G. duodenalis* which, because of the robust cyst transmission stage, can withstand standard chlorine treatment, as well as other water treatment processes [16]. Indeed, a plethora of studies has demonstrated that *Giardia* cysts occur frequently in aquatic environments [17].

Not surprisingly, giardiasis is the most reported waterborne parasitic disease in the EU/EEA, as confirmed by the 2017 Annual Epidemiologic Report of ECDC [5]. Although the disease is usually self-limiting, infected individuals can experience prolonged symptoms and/or treatment failure [18,19].

Here, we described the first documented human outbreak of giardiasis in Italy, which occurred in a municipality of the Bologna province, north-eastern Italy. The outbreak involved more than 200 individuals and extended over several months. Epidemiological investigations indicated tap water as the most probable source of infection, although *Giardia* contamination (cysts or DNA) in water samples was not demonstrated. The chemical-physical and microbiological parameters of the water samples were compliant with the European Water Regulation legislation, but this does not guarantee the absence of *Giardia* cysts. Due to the small (1 L) volumes of most water samples analysed, contamination with cyst numbers below the level of detection cannot be excluded.

The waterworks serves Municipality A as well as four other municipalities, but cases of giardiasis were only recorded in Municipality A. Although no spatial association was found during the exposure period between the location of plumbing maintenance work in Municipality A and the streets where giardiasis cases resided, several operations of the water supply network were documented during the estimated period of exposure. It is therefore possible that water contamination may have occurred during these operations. Based on this evidence, we believe that the outbreak meets the criteria for being considered strongly or, at least probably, associated with water [20].

Investigation of other risk factors showed that regular consumption of raw vegetables and fruits was associated with a decreased risk of giardiasis, in line with previous findings [21,22]. This may result from exposure to low levels of *Giardia* cysts present on these food items, which can trigger protective antibodies. However, a case–control study of risk factors for sporadic giardiasis in the UK identified eating lettuce as positively associated with the infection [23].

Finally, our findings indicate that drinking filtered tap water reduced, but did not eliminate, the risk of acquiring *Giardia* infection. This may be due to the fact that only tap water intended for consumption is filtered, while water used for other purposes (e.g. to rinse vegetables) is generally not. In addition, cases may have acquired infection by drinking tap water in places other than home.

Diagnosis of *Giardia* infection at ParaLab-Bo routinely relies on microscopy. As it was essential to reduce the time expended per sample while increasing the sensitivity, a sensitive and rapid antigen test [10] was rapidly implemented during the outbreak to replace microscopy. In line with previous studies [24], this test was of particular value in the outbreak setting, as the workload exceeded the laboratory’s capacity.

Molecular characterisation of *G. duodenalis* showed predominance of assemblage B (95%; 129/136 typed cases), although assemblage A was also identified. Moreover, the occurrence of three assemblage B genotypes and three assemblage A genotypes in patient samples suggests that genetically different cysts were present in the suspected vehicle of infection. The occurrence of multiple parasite genotypes in water is a common finding [25]. For example, during the large outbreak in Bergen, Norway, genetic analysis of 21 patient isolates consistently identified assemblage B, yet many genotypes were observed [26].

In Italy, investigation of wastewater treatment plants and surface waters demonstrated the presence of cysts of *G. duodenalis* assemblages A and B [27-29]. The high cyst count (10^3^–10^4^ per L) in raw wastewater of urban origin in these studies suggests an important circulation of the parasite in the population contributing to the wastewater flow. This seems to contrast with the low prevalence of giardiasis observed in the few studies conducted in Italy, and with a lack of officially reported cases. In fact, giardiasis is not included in the list of notifiable diseases in Italy and surveillance is not compulsory, therefore this parasitic infection remains under-diagnosed and under-reported. Similar discrepancies between research-derived data and official reports from 19 public health authorities of Eastern European countries has been recently reviewed [30].

This study has some limitations. In particular, it was difficult to ascertain the impact of possible recall biases during the investigation of risk factors by telephone interviews, or to ensure the absence of bias in the selection of controls for the case–control study.

## Conclusions

Our investigation indicates that passive surveillance of laboratory-confirmed cases did not allow for timely detection of an outbreak caused by a neglected pathogen, such as *G. duodenalis*. In turn, this highlights the need to increase awareness of giardiasis among GPs and paediatricians and to encourage routine diagnosis of the parasite in patients with persistent diarrhoea. A better appreciation of the health impact of giardiasis in Italy, as well as an improvement in the recording and reporting of cases, would contribute to progress in surveillance, including more timely recognition and management of outbreaks.
